# Bis(2,6-diamino­pyridin-1-ium) hexa­aqua­cobalt(II) disulfate dihydrate

**DOI:** 10.1107/S1600536810026693

**Published:** 2010-07-10

**Authors:** Mohammad T. M. Al-Dajani, Habibah A Wahab, Nornisah Mohamed, Jia Hao Goh, Hoong-Kun Fun

**Affiliations:** a School of Pharmaceutical Sciences, Universiti Sains Malaysia, 11800 USM, Penang, Malaysia; b X-ray Crystallography Unit, School of Physics, Universiti Sains Malaysia, 11800 USM, Penang, Malaysia

## Abstract

In the title compound, (C_5_H_8_N_3_)_2_[Co(H_2_O)_6_](SO_4_)_2_·2H_2_O, the complete complex cation is generated by crystallographic inversion symmetry, such that the Co^II^ cation is octa­hedrally coordinated by six water mol­ecules. The organic cation is essentially planar, with a maximum deviation of 0.013 (1) Å. In the crystal structure, the ions and mol­ecules are linked into a pseudo-layered three-dimensional supra­molecular network *via* O—H⋯O and N—H⋯O hydrogen bonds. Weak inter­molecular π–π inter­actions further stabilize the crystal structure [centroid–centroid distance = 3.5231 (4) Å].

## Related literature

For general background to and applications of 1,6-diamino­pyridinium ions, see: Abu Zuhri & Cox (1989 ▶); Inuzuka & Fujimoto (1990 ▶); Ma & Huang (2003 ▶); Patani & LaVoie (1996 ▶). For closely related hexa­aqua­cobalt(II) structures, see: Li *et al.* (2004 ▶); Pan *et al.* (2003 ▶). For closely related pyridinium structures, see: Al-Dajani, Abdallah *et al.* (2009 ▶, 2010 ▶); Al-Dajani, Salhin *et al.* (2009 ▶). For the stability of the temperature controller used for the data collection, see: Cosier & Glazer (1986 ▶).
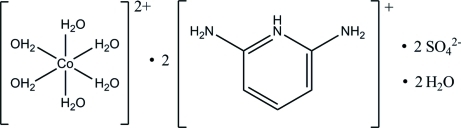


## Experimental

### 

#### Crystal data

(C_5_H_8_N_3_)_2_[Co(H_2_O)_6_](SO_4_)_2_·2H_2_O
*M* *_r_* = 615.47
Orthorhombic, 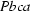

*a* = 6.6219 (1) Å
*b* = 14.4347 (2) Å
*c* = 24.7590 (3) Å
*V* = 2366.59 (6) Å^3^
*Z* = 4
Mo *K*α radiation
μ = 0.99 mm^−1^
*T* = 100 K
0.35 × 0.31 × 0.21 mm

#### Data collection

Bruker SMART APEXII CCD diffractometer
Absorption correction: multi-scan (*SADABS*; Bruker, 2009 ▶) *T*_min_ = 0.725, *T*_max_ = 0.821
86569 measured reflections
6333 independent reflections
5758 reflections with *I* > 2σ(*I*)
*R*_int_ = 0.030

#### Refinement

*R*[*F* ^2^ > 2σ(*F* ^2^)] = 0.022
*wR*(*F* ^2^) = 0.064
*S* = 1.08
6333 reflections
224 parameters
All H-atom parameters refined
Δρ_max_ = 0.60 e Å^−3^
Δρ_min_ = −0.38 e Å^−3^

### 

Data collection: *APEX2* (Bruker, 2009 ▶); cell refinement: *SAINT* (Bruker, 2009 ▶); data reduction: *SAINT*; program(s) used to solve structure: *SHELXTL* (Sheldrick, 2008 ▶); program(s) used to refine structure: *SHELXTL*; molecular graphics: *SHELXTL*; software used to prepare material for publication: *SHELXTL* and *PLATON* (Spek, 2009 ▶).

## Supplementary Material

Crystal structure: contains datablocks global, I. DOI: 10.1107/S1600536810026693/hb5536sup1.cif

Structure factors: contains datablocks I. DOI: 10.1107/S1600536810026693/hb5536Isup2.hkl

Additional supplementary materials:  crystallographic information; 3D view; checkCIF report

## Figures and Tables

**Table 1:** Selected bond lengths (Å)

Co1—O1*W*	2.0801 (5)
Co1—O2*W*	2.0985 (5)
Co1—O3*W*	2.1064 (5)

**Table 2:** Hydrogen-bond geometry (Å, °)

*D*—H⋯*A*	*D*—H	H⋯*A*	*D*⋯*A*	*D*—H⋯*A*
O1*W*—H1*W*1⋯O1^i^	0.848 (14)	1.863 (15)	2.7088 (7)	175.4 (13)
O1*W*—H2*W*1⋯O4*W*^ii^	0.840 (13)	1.948 (13)	2.7853 (8)	174.2 (13)
O2*W*—H1*W*2⋯O2	0.800 (14)	1.923 (14)	2.7217 (8)	176.3 (14)
O2*W*—H2*W*2⋯O3^iii^	0.815 (14)	2.025 (13)	2.8352 (8)	172.7 (15)
O3*W*—H1*W*3⋯O4*W*^i^	0.836 (15)	1.898 (15)	2.7318 (8)	175.1 (13)
O3*W*—H2*W*3⋯O3^iv^	0.805 (15)	1.991 (15)	2.7928 (8)	173.6 (14)
O4*W*—H1*W*4⋯O2	0.863 (17)	1.910 (17)	2.7643 (8)	169.9 (16)
O4*W*—H2*W*4⋯O3^i^	0.784 (15)	1.994 (15)	2.7157 (7)	152.8 (18)
N1—H1*N*1⋯O1	0.883 (13)	2.005 (12)	2.8412 (7)	157.6 (12)
N2—H2*N*2⋯O2^v^	0.810 (15)	2.424 (15)	3.1769 (9)	155.1 (13)
N3—H1*N*3⋯O1	0.851 (13)	2.347 (13)	3.0660 (7)	142.6 (11)
N2—H1*N*2⋯O4	0.776 (15)	2.162 (15)	2.8977 (9)	158.5 (14)
N3—H2*N*3⋯O4^vi^	0.844 (14)	2.079 (14)	2.9155 (8)	171.0 (12)

Symmetry codes: (i) 
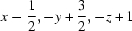
; (ii) 
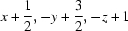
; (iii) 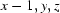; (iv) 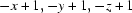; (v) 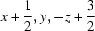; (vi) 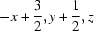.

**Fig. 1.**
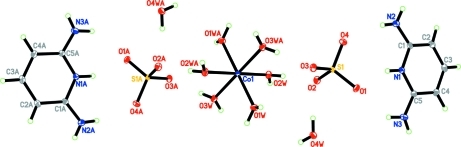
The molecular structure of (I), showing 50% probability displacement ellipsoids for non-H atoms. The suffix A corresponds to the symmetry code [-x+1, -y+1, -z+1].

**Fig. 2.**
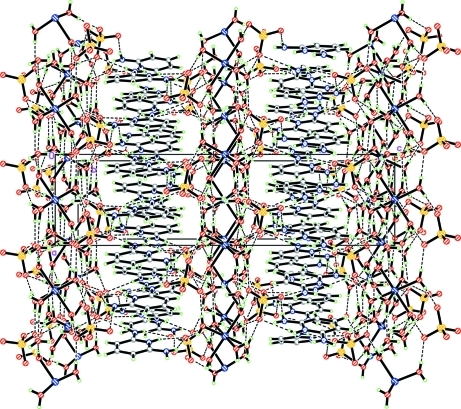
The crystal structure of (I), viewed along the b axis, showing the three-dimensional supramolecular structure. Intermolecular interactions have been shown as dashed lines.

## supplementary crystallographic information

### Comment

Generally 1,6-diaminopyridinium has an important role in the preparation of aromatic azo dyes, the subject of many polarographic investigations (Abu Zuhri & Cox, 1989). It also exhibits amino-imino tautomerization property (Inuzuka & Fujimoto, 1990). Molecules containing pyridyl moiety exhibit biological activity and low toxicity (Patani & LaVoie, 1996; Ma & Huang, 2003).

The asymmetric unit of the title complex comprises of half of hexaaquacobalt(II) cation, a protonated 2,6-diaminopyridin-1-ium cation, a sulphate anion and a water molecule of crystallization. The complete complex (Fig. 1) is generated by the crystallographic inversion center [symmetry code of atoms labelled with suffix A: -x+1, -y+1, -z+1]. Within the metal complex cation [Co(H_2_O)_6_]^2+^, the Co^II^ ion is coordinated by six water molecules at the vertices of the almost ideal octahedron. The Co-O bond lengths range from 2.0801 (5) to 2.1064 (5) Å and the O—Co—O angles span the ranges of 87.50 (2)–92.50 (2)° and 179.999 (1)–180.00 (3)°. The 2,6-diaminopyridinium organic cation (C1-C5/N1-N3) is essentially planar, with a maximum deviation of -0.013 (1) Å at atom C5. Comparing to the unprotonated structure (Al-Dajani, Salhin *et al.*, 2009), protonation at atom N1 has lead to a slight increase in the C1—N1—C5 angle to 123.55 (5)°. The geometric parameters are consistent to those observed in closely related hexaaquacobalt(II) (Pan *et al.*, 2003; Li *et al.*, 2004) and 1,6-diaminopyridinium (Al-Dajani, Abdallah *et al.*, 2009,2010; Al-Dajani, Salhin *et al.*, 2009) structures.

The crystal structure is mainly stabilized by a network of O—H···O and N—H···O hydrogen bonds (Table 2). In this network, the water molecule O atoms and organic N atoms act as donors whereas the sulphate O atoms provide the most extensive part as acceptors. A three-dimensional supramolecular structure (Fig. 2) is built up in such an arrangement that the 2,6-diaminopyridinium organic layers are sandwiched between layers formed through the remaining ions and water molecules. The crystal structure is further stabilized by weak intermolecular *Cg*1···*Cg*1 interactions [*Cg*1···*Cg*1 = 3.5231 (4) Å; symmetry codes: x-1/2, y, -z+3/2 and x+1/2, y, -z+3/2] where *Cg*1 is the centroid of C1-C5/N1 pyridine ring.

### Experimental

In a round bottom flask was added with stirring 1,4-dioxane (25 ml), 2,6-diaminopyridine (0.02 mol, 2.2 g) and CoSO_4_.7H_2_O (0.01 mol, 2.8 g) dissolved in water. The concoction was refluxed for 24 h and a red solution was then formed. Red blocks of (I) were formed overnight at room temperature. The filtrate was washed with 1,4-dioxane and dried at 333 K.

### Refinement

All H-atoms were located from difference Fourier map and allowed to refine freely [ranges of C—H = 0.938 (13)–0.980 (13) Å, N—H = 0.776 (15)–0.884 (13) Å and O—H = 0.784 (16)—0.863 (17) Å].

### Crystal data

**Table:**

(C_5_H_8_N_3_)_2_[Co(H_2_O)_6_](SO_4_)_2_·2H_2_O	*F*(000) = 1284
*M**_r_* = 615.47	*D*_x_ = 1.727 Mg m^−^^3^
Orthorhombic, *P**b**c**a*	Mo *K*α radiation, λ = 0.71073 Å
Hall symbol: -P 2ac 2ab	Cell parameters from 9901 reflections
*a* = 6.6219 (1) Å	θ = 3.5–37.6°
*b* = 14.4347 (2) Å	µ = 0.99 mm^−^^1^
*c* = 24.7590 (3) Å	*T* = 100 K
*V* = 2366.59 (6) Å^3^	Block, red
*Z* = 4	0.35 × 0.31 × 0.21 mm

### Data collection

**Table:**

Bruker SMART APEXII CCD diffractometer	6333 independent reflections
Radiation source: fine-focus sealed tube	5758 reflections with *I* > 2σ(*I*)
graphite	*R*_int_ = 0.030
φ and ω scans	θ_max_ = 37.7°, θ_min_ = 1.6°
Absorption correction: multi-scan (*SADABS*; Bruker, 2009)	*h* = −11→11
*T*_min_ = 0.725, *T*_max_ = 0.821	*k* = −24→24
86569 measured reflections	*l* = −42→42

### Refinement

**Table:**

Refinement on *F*^2^	Primary atom site location: structure-invariant direct methods
Least-squares matrix: full	Secondary atom site location: difference Fourier map
*R*[*F*^2^ > 2σ(*F*^2^)] = 0.022	Hydrogen site location: inferred from neighbouring sites
*wR*(*F*^2^) = 0.064	All H-atom parameters refined
*S* = 1.08	*w* = 1/[σ^2^(*F*_o_^2^) + (0.0322*P*)^2^ + 0.6554*P*] where *P* = (*F*_o_^2^ + 2*F*_c_^2^)/3
6333 reflections	(Δ/σ)_max_ = 0.001
224 parameters	Δρ_max_ = 0.60 e Å^−^^3^
0 restraints	Δρ_min_ = −0.38 e Å^−^^3^

### Special details

**Table:**

Experimental. The crystal was placed in the cold stream of an Oxford Cryosystems Cobra open-flow nitrogen cryostat (Cosier & Glazer, 1986) operating at 100.0 (1)K.
Geometry. All esds (except the esd in the dihedral angle between two l.s. planes) are estimated using the full covariance matrix. The cell esds are taken into account individually in the estimation of esds in distances, angles and torsion angles; correlations between esds in cell parameters are only used when they are defined by crystal symmetry. An approximate (isotropic) treatment of cell esds is used for estimating esds involving l.s. planes.
Refinement. Refinement of F^2^ against ALL reflections. The weighted R-factor wR and goodness of fit S are based on F^2^, conventional R-factors R are based on F, with F set to zero for negative F^2^. The threshold expression of F^2^ > 2sigma(F^2^) is used only for calculating R-factors(gt) etc. and is not relevant to the choice of reflections for refinement. R-factors based on F^2^ are statistically about twice as large as those based on F, and R- factors based on ALL data will be even larger.

### Fractional atomic coordinates and isotropic or equivalent isotropic displacement parameters (Å^2^)

**Table:**

	*x*	*y*	*z*	*U*_iso_*/*U*_eq_	
Co1	0.5000	0.5000	0.5000	0.00813 (3)	
O1W	0.60030 (8)	0.62537 (4)	0.46843 (2)	0.01288 (8)	
O2W	0.37213 (8)	0.57254 (4)	0.56500 (2)	0.01253 (8)	
O3W	0.23481 (8)	0.51148 (4)	0.45350 (2)	0.01281 (9)	
O4W	0.50958 (8)	0.83091 (4)	0.53640 (2)	0.01281 (9)	
S1	0.86829 (2)	0.696844 (10)	0.616698 (6)	0.00760 (3)	
O1	0.93408 (8)	0.79469 (3)	0.62001 (2)	0.01200 (8)	
O2	0.64851 (8)	0.69401 (4)	0.60506 (2)	0.01254 (8)	
O3	0.97898 (8)	0.64929 (4)	0.57237 (2)	0.01158 (8)	
O4	0.90760 (9)	0.65018 (4)	0.66828 (2)	0.01417 (9)	
N1	0.89603 (9)	0.85415 (4)	0.72893 (2)	0.00943 (8)	
N2	0.97396 (10)	0.72061 (4)	0.77643 (3)	0.01420 (10)	
N3	0.82794 (10)	0.97979 (4)	0.67371 (2)	0.01328 (10)	
C1	0.91746 (10)	0.81030 (4)	0.77745 (2)	0.00979 (9)	
C2	0.88017 (10)	0.86024 (5)	0.82461 (3)	0.01229 (10)	
C3	0.82632 (11)	0.95288 (5)	0.82032 (3)	0.01297 (10)	
C4	0.80547 (11)	0.99626 (5)	0.77070 (3)	0.01179 (10)	
C5	0.84005 (9)	0.94478 (4)	0.72390 (2)	0.00965 (9)	
H1W1	0.542 (2)	0.6492 (10)	0.4413 (6)	0.030 (4)*	
H2W1	0.724 (2)	0.6368 (10)	0.4648 (6)	0.028 (3)*	
H1W2	0.451 (2)	0.6080 (10)	0.5781 (6)	0.025 (3)*	
H2W2	0.264 (2)	0.5990 (10)	0.5680 (6)	0.030 (4)*	
H1W3	0.160 (2)	0.5580 (11)	0.4558 (6)	0.029 (4)*	
H2W3	0.165 (2)	0.4675 (11)	0.4463 (6)	0.028 (3)*	
H1W4	0.559 (3)	0.7850 (12)	0.5544 (6)	0.036 (4)*	
H2W4	0.514 (2)	0.8192 (13)	0.5055 (6)	0.032 (4)*	
H1N1	0.923 (2)	0.8224 (9)	0.6993 (5)	0.021 (3)*	
H2N2	1.014 (2)	0.6963 (10)	0.8039 (6)	0.023 (3)*	
H1N3	0.836 (2)	0.9442 (9)	0.6464 (5)	0.021 (3)*	
H1N2	0.983 (2)	0.6940 (10)	0.7493 (6)	0.023 (3)*	
H2N3	0.769 (2)	1.0311 (10)	0.6695 (5)	0.022 (3)*	
H2	0.888 (2)	0.8281 (9)	0.8575 (5)	0.024 (3)*	
H3	0.794 (2)	0.9854 (9)	0.8525 (5)	0.017 (3)*	
H4	0.768 (2)	1.0617 (9)	0.7676 (5)	0.021 (3)*	

### Atomic displacement parameters (Å^2^)

**Table:**

	*U*^11^	*U*^22^	*U*^33^	*U*^12^	*U*^13^	*U*^23^
Co1	0.00772 (5)	0.00786 (5)	0.00880 (5)	−0.00035 (4)	−0.00063 (4)	0.00026 (3)
O1W	0.01163 (19)	0.0125 (2)	0.0145 (2)	−0.00216 (16)	−0.00227 (17)	0.00427 (15)
O2W	0.01003 (19)	0.0139 (2)	0.01366 (19)	−0.00017 (16)	−0.00004 (16)	−0.00345 (16)
O3W	0.01056 (19)	0.01071 (19)	0.0172 (2)	0.00053 (16)	−0.00386 (17)	−0.00187 (15)
O4W	0.0138 (2)	0.0137 (2)	0.01087 (19)	0.00064 (16)	−0.00068 (16)	−0.00023 (16)
S1	0.00845 (6)	0.00744 (6)	0.00691 (5)	−0.00042 (4)	0.00023 (4)	−0.00030 (4)
O1	0.0154 (2)	0.00811 (18)	0.01252 (19)	−0.00296 (16)	0.00154 (17)	−0.00110 (14)
O2	0.00834 (18)	0.0135 (2)	0.0158 (2)	−0.00022 (15)	−0.00093 (16)	−0.00211 (16)
O3	0.01201 (19)	0.01219 (19)	0.01052 (18)	0.00079 (15)	0.00229 (15)	−0.00298 (15)
O4	0.0201 (2)	0.0137 (2)	0.00873 (17)	−0.00068 (18)	−0.00182 (17)	0.00294 (15)
N1	0.0103 (2)	0.0097 (2)	0.00826 (19)	0.00011 (16)	−0.00031 (16)	−0.00037 (15)
N2	0.0180 (3)	0.0110 (2)	0.0136 (2)	0.00237 (19)	−0.0038 (2)	0.00052 (18)
N3	0.0147 (2)	0.0138 (2)	0.0113 (2)	0.00146 (19)	−0.00063 (19)	0.00307 (17)
C1	0.0089 (2)	0.0109 (2)	0.0096 (2)	−0.00068 (18)	−0.00122 (18)	0.00055 (17)
C2	0.0127 (2)	0.0153 (3)	0.0089 (2)	0.0001 (2)	−0.00031 (19)	−0.00021 (18)
C3	0.0119 (2)	0.0157 (3)	0.0113 (2)	0.0000 (2)	0.0004 (2)	−0.00380 (19)
C4	0.0112 (2)	0.0108 (2)	0.0134 (2)	0.00028 (19)	0.0003 (2)	−0.00217 (18)
C5	0.0080 (2)	0.0101 (2)	0.0109 (2)	−0.00041 (18)	−0.00031 (18)	0.00074 (17)

### Geometric parameters (Å, °)

**Table:**

Co1—O1W^i^	2.0801 (5)	S1—O3	1.4875 (5)
Co1—O1W	2.0801 (5)	N1—C1	1.3652 (8)
Co1—O2W^i^	2.0985 (5)	N1—C5	1.3654 (8)
Co1—O2W	2.0985 (5)	N1—H1N1	0.884 (13)
Co1—O3W^i^	2.1064 (5)	N2—C1	1.3478 (9)
Co1—O3W	2.1064 (5)	N2—H2N2	0.810 (14)
O1W—H1W1	0.848 (16)	N2—H1N2	0.776 (15)
O1W—H2W1	0.841 (16)	N3—C5	1.3438 (8)
O2W—H1W2	0.799 (15)	N3—H1N3	0.851 (14)
O2W—H2W2	0.817 (16)	N3—H2N3	0.843 (15)
O3W—H1W3	0.836 (16)	C1—C2	1.3942 (9)
O3W—H2W3	0.806 (16)	C2—C3	1.3881 (10)
O4W—H1W4	0.863 (17)	C2—H2	0.938 (13)
O4W—H2W4	0.784 (16)	C3—C4	1.3859 (10)
S1—O4	1.4672 (5)	C3—H3	0.949 (13)
S1—O1	1.4803 (5)	C4—C5	1.3953 (9)
S1—O2	1.4842 (5)	C4—H4	0.980 (13)
			
O1W^i^—Co1—O1W	180.0	O4—S1—O3	110.06 (3)
O1W^i^—Co1—O2W^i^	89.02 (2)	O1—S1—O3	109.64 (3)
O1W—Co1—O2W^i^	90.98 (2)	O2—S1—O3	109.10 (3)
O1W^i^—Co1—O2W	90.98 (2)	C1—N1—C5	123.55 (5)
O1W—Co1—O2W	89.02 (2)	C1—N1—H1N1	117.9 (9)
O2W^i^—Co1—O2W	180.0	C5—N1—H1N1	118.5 (9)
O1W^i^—Co1—O3W^i^	89.56 (2)	C1—N2—H2N2	119.5 (10)
O1W—Co1—O3W^i^	90.44 (2)	C1—N2—H1N2	120.9 (11)
O2W^i^—Co1—O3W^i^	92.50 (2)	H2N2—N2—H1N2	119.0 (15)
O2W—Co1—O3W^i^	87.50 (2)	C5—N3—H1N3	120.2 (9)
O1W^i^—Co1—O3W	90.44 (2)	C5—N3—H2N3	118.2 (9)
O1W—Co1—O3W	89.56 (2)	H1N3—N3—H2N3	117.5 (13)
O2W^i^—Co1—O3W	87.50 (2)	N2—C1—N1	117.24 (6)
O2W—Co1—O3W	92.50 (2)	N2—C1—C2	124.16 (6)
O3W^i^—Co1—O3W	180.0	N1—C1—C2	118.60 (6)
Co1—O1W—H1W1	120.3 (10)	C3—C2—C1	118.66 (6)
Co1—O1W—H2W1	121.4 (10)	C3—C2—H2	123.9 (8)
H1W1—O1W—H2W1	106.3 (14)	C1—C2—H2	117.4 (8)
Co1—O2W—H1W2	111.5 (11)	C4—C3—C2	121.91 (6)
Co1—O2W—H2W2	131.1 (10)	C4—C3—H3	120.0 (8)
H1W2—O2W—H2W2	103.8 (14)	C2—C3—H3	118.0 (8)
Co1—O3W—H1W3	121.3 (10)	C3—C4—C5	118.63 (6)
Co1—O3W—H2W3	122.5 (11)	C3—C4—H4	122.0 (8)
H1W3—O3W—H2W3	108.0 (16)	C5—C4—H4	119.4 (8)
H1W4—O4W—H2W4	109.0 (17)	N3—C5—N1	117.44 (6)
O4—S1—O1	109.73 (3)	N3—C5—C4	123.90 (6)
O4—S1—O2	109.29 (3)	N1—C5—C4	118.63 (6)
O1—S1—O2	109.01 (3)		
			
C5—N1—C1—N2	−179.89 (6)	C2—C3—C4—C5	−0.05 (11)
C5—N1—C1—C2	0.09 (10)	C1—N1—C5—N3	178.90 (6)
N2—C1—C2—C3	178.85 (7)	C1—N1—C5—C4	0.99 (10)
N1—C1—C2—C3	−1.13 (10)	C3—C4—C5—N3	−178.76 (7)
C1—C2—C3—C4	1.12 (11)	C3—C4—C5—N1	−0.99 (10)

Symmetry codes: (i) −*x*+1, −*y*+1, −*z*+1.

### Hydrogen-bond geometry (Å, °)

**Table:**

*D*—H···*A*	*D*—H	H···*A*	*D*···*A*	*D*—H···*A*
O1W—H1W1···O1^ii^	0.848 (14)	1.863 (15)	2.7088 (7)	175.4 (13)
O1W—H2W1···O4W^iii^	0.840 (13)	1.948 (13)	2.7853 (8)	174.2 (13)
O2W—H1W2···O2	0.800 (14)	1.923 (14)	2.7217 (8)	176.3 (14)
O2W—H2W2···O3^iv^	0.815 (14)	2.025 (13)	2.8352 (8)	172.7 (15)
O3W—H1W3···O4W^ii^	0.836 (15)	1.898 (15)	2.7318 (8)	175.1 (13)
O3W—H2W3···O3^i^	0.805 (15)	1.991 (15)	2.7928 (8)	173.6 (14)
O4W—H1W4···O2	0.863 (17)	1.910 (17)	2.7643 (8)	169.9 (16)
O4W—H2W4···O3^ii^	0.784 (15)	1.994 (15)	2.7157 (7)	152.8 (18)
N1—H1N1···O1	0.883 (13)	2.005 (12)	2.8412 (7)	157.6 (12)
N2—H2N2···O2^v^	0.810 (15)	2.424 (15)	3.1769 (9)	155.1 (13)
N3—H1N3···O1	0.851 (13)	2.347 (13)	3.0660 (7)	142.6 (11)
N2—H1N2···O4	0.776 (15)	2.162 (15)	2.8977 (9)	158.5 (14)
N3—H2N3···O4^vi^	0.844 (14)	2.079 (14)	2.9155 (8)	171.0 (12)

Symmetry codes: (ii) *x*−1/2, −*y*+3/2, −*z*+1; (iii) *x*+1/2, −*y*+3/2, −*z*+1; (iv) *x*−1, *y*, *z*; (i) −*x*+1, −*y*+1, −*z*+1; (v) *x*+1/2, *y*, −*z*+3/2; (vi) −*x*+3/2, *y*+1/2, *z*.
